# Probabilistic Movement Models Show that Postural Control Precedes and Predicts Volitional Motor Control

**DOI:** 10.1038/srep28455

**Published:** 2016-06-22

**Authors:** Elmar Rueckert, Jernej Čamernik, Jan Peters, Jan Babič

**Affiliations:** 1 Intelligent Autonomous Systems Lab, Technische Universität Darmstadt, 64289, Germany; 2 Department for Automation, Biocybernetics and Robotics, Jožef Stefan Institute, Ljubljana, SI-1000, Slovenia; 3 Robot Learning Group, Max-Planck Institute for Intelligent Systems, Tuebingen, 72076, Germany

## Abstract

Human motor skill learning is driven by the necessity to adapt to new situations. While supportive contacts are essential for many tasks, little is known about their impact on motor learning. To study the effect of contacts an innovative full-body experimental paradigm was established. The task of the subjects was to reach for a distant target while postural stability could only be maintained by establishing an additional supportive hand contact. To examine adaptation, non-trivial postural perturbations of the subjects’ support base were systematically introduced. A novel probabilistic trajectory model approach was employed to analyze the correlation between the motions of both arms and the trunk. We found that subjects adapted to the perturbations by establishing target dependent hand contacts. Moreover, we found that the trunk motion adapted significantly faster than the motion of the arms. However, the most striking finding was that observations of the initial phase of the left arm or trunk motion (100–400 ms) were sufficient to faithfully predict the complete movement of the right arm. Overall, our results suggest that the goal-directed arm movements determine the supportive arm motions and that the motion of heavy body parts adapts faster than the light arms.

Most of our every day motor skills involve strict control of postural stability in parallel to the execution of the primary motor task. A great deal of these tasks also require additional supportive hand contacts beside the feet that are in contact with the ground. An example task is the reaching for a glass on the highest kitchen shelf when we typically have to use the other hand to support the body by leaning on the kitchen counter. To successfully perform such a reaching motion and to simultaneously ensure the postural stability, the motion of the body and both arms have to be perfectly synchronized and coordinated. Till now it is unclear how this coordination develops or adapts during skill learning.

In general, supportive hand contacts increase the stability during balancing and complement humans’ sophisticated motor abilities. Already *light touch* fingertip contacts provide sufficient feedback to enhance postural stability^1^,^2^,^3^,^4^. Notable mechanical support is gained by holding onto a handrail, which enables humans to generate contact forces to better counteract perturbations^5^,^6^. A notable exception to these static contacts studies are fall prevention experiments. It was shown that humans prefer reach-to-grasp strategies to rapid stepping with age^7^,^8^. All these works underline the importance of contacts but cannot answer the posed question of how the coordination of supportive contacts and primary tasks develops or adapts.

Like the ability to balance, the utilization of supportive hand contacts has to be learned^9^,^10^. In the early childhood, learning of postural balance is tightly intertwined with supportive hand motion. At eight months infants already learn how to balance on hands and knees during crawling; at ten months they start walking with support from hanging on a table or a couch; and at about one year infants already know how to balance on two feet and walk independently across the room^11^,^12^. While it is known that people develop optimal motor control strategies both for postural stability and manipulation^13^,^14^,^15^,^16^, it is unclear how these two distinctive motor tasks relate to each other and how their relationship affect the *learning* of novel motor skills or when re-establishing motor abilities in novel environments. A thorough understanding of these issues is of particular interest for the field of rehabilitation, where correlations between the primary motor tasks and the underlying supportive motor actions could be exploited in progress monitoring and novel pre-tests of motor dysfunctions^17^. Such pre-tests promise to be less sensitive to factors like stress or sleep deprivation. However, to approach that goal we need to identify such correlations if existing.

Many of previous studies either focus on control of postural stability^18^,^19^,^20^,^21^, study adaptation of arm reaching in confined lab environments^22^,^23^,^24^,^25^,^26^,^27^,^28^,^29^, or study situations when postural stability and arm reaching are only indirectly related^2^,^6^,^30^,^31^,^32^. In our study we propose an innovative full-body experimental paradigm (Fig. 1A) that extends current experimental methods to a more ecological setting where postural stability and manipulation skills are tightly interrelated and interdependent to each other.

We hypothesize that the arm reaching motions determine the supportive hand contact strategies and that both motor tasks adapt during training. In particular, we assume that these two motor tasks are correlated which is reflected in synchronized motor executions and a significant correlation between target locations and supportive hand contacts. To effectively elaborate on these hypotheses we designed an experimental paradigm where we asked 20 healthy subjects to reach with their right arm for a target displayed on a screen while using their left arm to maintain postural stability by leaning on a table in front of them. Non-trivial postural perturbations of the subjects’ support base were systematically introduced to examine adaptation and a novel probabilistic trajectory model approach was employed to analyze the correlations between the supportive arm motion and the motion of the arm to reach for the distant target.

## Results

The experimental setup involved a 6 degrees-of-freedom Stewart platform on which human subjects stood and performed the arm-reaching movements. The sum of the anteroposterior components of the left and right hand displacements during the reaching was used to generate (or not, depending on the condition) perturbations in the mediolateral direction; the forward motion of hands caused a leftward displacement of the platform as depicted in Supplementary Figure S5. The subjects performed arm-reaching movements (called trials) in three types of conditions: perturbed (P), unperturbed (UP) and catch (C). In the unperturbed condition, the platform remained still while in the perturbed condition the platform induced perturbations. The catch condition was almost the same as the perturbed case. However, for some randomly selected squat-to-stand trials, the perturbation was turned off without the knowledge of the subjects.

**Figure 1:**
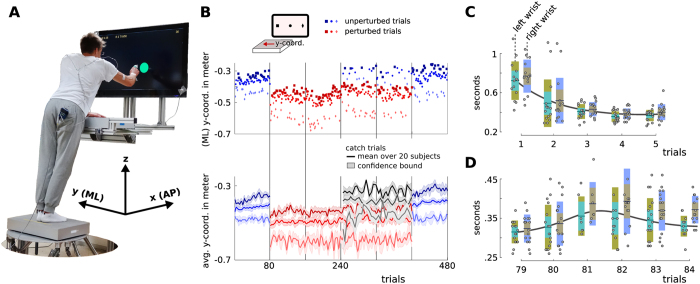
Experiment, target dependent contacts and synchronized arm motions. (**A**) Experimental setting. (**B**) The top row shows contact locations for a single representative subject and the bottom row shows the mean and the confidence bound over all 20 participants. The first 80 trials and the last 80 trials are unperturbed sessions. Catch trials were initiated during trials 240 to 400 and are denoted by the black lines in (**B**). (**C**) Illustration of the movement onsets of the wrists for the first five trials. (**D**) Movement onsets when transitioning to the perturbed session (trials 79 and 80 are still unperturbed).

To initiate the reaching movements of the subject, a target was presented on the screen at one out of three possible locations. The target appeared randomly at the left, the center or the right side on a horizontal bar at a random interval of one to three seconds after the previous trial. We motivated the subjects through a monetary reward that was proportional to the time needed to reach for the target. This reward was displayed after the target was reached. The number zero was displayed if the trial time exceeded two seconds. In our results, we focused on the y-coordinate of the contact locations as it corresponded to the direction of the translational perturbation. This is indicated by the arrow labeled by *y* in Fig. 1.

### Subjects chose task dependent contact locations

Analysis of variance (ANOVA) showed a significant effect in the left hand contact location between sessions when the subjects were perturbed and unperturbed during reaching toward targets displayed on screen, *F*(1,19) = 113.632, *p* < 0.001, 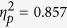.

The post-hoc analysis showed that the left hand contact location was significantly different between reaching toward all three distinctly positioned targets in perturbed, *t*(19) = 6.85to11.58, *p* < 0.001, and in unperturbed trials, *t*(19) = 6.78–12.21, *p* < 0.001.

There were also significant differences between means of contact locations for each target between perturbed and unperturbed trials (target one, *t*(19) = 10.07, *p* < 0.001, *d* = 2.01; target two, *t*(19) = 10.22, *p* < 0.001, *d* = 2.38; target three, *t*(19) = 11.51, *p* < 0.001, *d* = 3.25), see Supplementary Figure S1.

Further, we analyzed individual sessions of unperturbed, perturbed and catch trials. We compared them with each other to determine whether there are any differences between contact locations and for which target positions they apply.

The first 80 trials out of 480 training trials were unperturbed. We found that there was a significant effect of target locations on the supportive contact locations in these first 80 trials, *F*(1.11,21.14) = 104.61, *p* < 0.001, 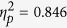, see Fig. 1B. Post-hoc tests using the Bonferroni correction^33^ revealed that there was significant difference in chosen supportive contact location between all three targets (*t*(19) = 6.92 to11.66, *p* < 0.001).

After the initial training phase, all subjects had to adapt to translational perturbations of the support base. Note that to compare the contact locations and the marker trajectories of perturbed and unperturbed trials, we corrected all sensor readings by the motion of the moving base. We refer to this correction as the *subject frame*. Later we will introduce *shoulder frames* to separate adaptation in the trunk and in the arms. Again, we found that there was a significant effect of the target location on the contact location over all perturbed trials, *F*(1.33,25.27) = 116.39, *p* < 0.001, 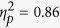, see trials 81to240 in Fig. 1B. Post-hoc tests showed a significant difference in the chosen supportive contact location among all three targets (*t*(19) = 6.85to11.58, *p* < 0.001).

Consolidation of the postural control strategies was tested through randomly initiating unperturbed trials (catch trials). In the last phase of perturbed trials (trials 320 to 400), where adaptation can be assumed to be converged, the perturbation was deactivated for 30 random catch trials. When comparing initial training trials with catch trials post-hoc tests showed no significant difference for all three targets, target one (*t*(18) = −1.99, *p* = 0.062, *d* = −0.74), target two (*t*(18) = −3.28, *p* = 0.004, *d* = −1) and target three (*t*(18) = −1.03, *p* = 0.315, *d* = −0.39). Comparison of perturbed and catch trials showed significant differences in contact locations for all three target locations (*t*(18) = −11.24 to −12.97, *p* < 0.001, *d* = −2.44 to −1.93). For the washout, no significant difference was observed between contact locations for individual targets when comparing catch and final trials 401 to 480 (*t*(18) = −1.59 to 1.28, *p* = 0.13 to 0.22, *d* = −1 to −0.39).

In summary, we found that subjects chose distinct contact locations dependent on the target location on the screen. Catch trial tests revealed that the participants learned specialized control strategies for unperturbed and perturbed conditions. However, whether the distinct contacts result from trunk or left arm adaptations can not be answered from looking at contact locations only. That requires a more detailed analysis of the temporal profiles for which we will introduce a probabilistic trajectory model.

### Supportive and goal-directed movements synchronize

Decreasing reaction times are an indicator for adaption of a pre-processing phase^34^. In line with this, we found that the movement onsets of the left and the right wrist synchronize within 3–5 trials in the unperturbed training phase (left wrist: 323 ± 44(*SD*) ms, right wrist: 335 ± 46 ms) and when the perturbations are experienced for the first time (left wrist: 333 ± 48 ms, right wrist: 342 ± 49 ms). The movement onsets of all subjects in the first five unperturbed trials are shown in Fig. 1C. In Fig. 1D onsets of the last two unperturbed trials (79 and 80) and of the first four perturbed trials are shown.

No significant lead or lag of the supportive left arm motion over the right arm reaching movements was found in the data. This is shown in an illustration of movement onsets of all subjects in Supplementary Figure S2.

### Modeling joint distributions over limb trajectories

Reaction times are sensitive to signal noise, need to be verified through visual inspection and allow only for a limited view on the functional mechanisms during skill learning. As an alternative to reaction time studies, we propose to analyze adaptation on a trajectory basis. For that we developed a probabilistic trajectory model (PTM) that encodes a joint distribution over multiple limb trajectories and over multiple coordinates like x, y, z components of three-dimensional marker data. Here, we give a brief summary of PTMs and for a precise mathematical definition we refer the reader to the Methods section.

**Figure 2:**
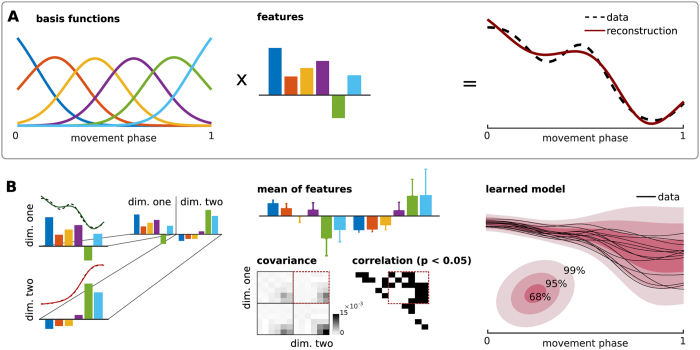
Probabilistic model of trajectories, from a feature space (left column) to trajectories (right column). (**A**) Generative model. Equally spaced (radial) basis functions are amplitude scaled by a feature vector to approximate a one-dimensional trajectory. A movement phase substitutes time to model trajectories of different lengths. In the inverse direction, feature vectors are computed given the input trajectories using, e.g., standard linear regression techniques or iterative variational approaches like expectation-maximization. (**B**) Learning the correlations between multi-dimensional input trajectories. Feature vectors computed from multiple input trajectories are concatenated and the mean and the covariance are computed from multiple trials (or subjects). The learned correlation in the center panel is the key features for computing predictions from partial observations. The distribution over trajectories is shown in the right panel.

The main feature of the model is that it captures the correlations between individual input dimensions. The model builds on a linear function approximator using radial basis functions with fixed means and variances. The amplitudes of the basis functions are scaled by learnable feature weights. Now both, the basis functions and the weights, form a linear generative model of a trajectory, i.e., **τ** = **Φ*****w*** (with the trajectory ***τ*** = [*y*_1_, *y*_2_,..., *y*_*t*_], time-varying observations *y*_*t*_, basis functions in **Φ** and feature weights ***w***). Figure 2A shows the encoding of a single trajectory using classical radial basis functions. Note that the model is linear in the feature space, however it can capture non-linear dependencies in the trajectory space (through non-linear basis functions). The model’s complexity is controlled through the number of basis functions. For the reaching experiments ten Gaussian distributions per dimension were found to be sufficient, see Supplementary Figure S3.

The feature vector ***w*** can be learned in the most simple case through standard linear regression or in more sophisticated models through one of the many existing variational inference methods^35^.

A PTM encodes multiple input dimensions through a concatenated feature vector (a two-dimensional example is illustrated in Fig. 2B). This concatenated feature vector scales the contribution of an *extended* basis function matrix. Thus, the only difference to standard radial basis functions is a clever arrangement of basis functions and feature vectors to represent multiple input dimensions in one model.

To model a distribution over trajectories the mean and the covariance over multiple trials are computed from the inferred feature vectors (e.g., through linear regression). An example is shown in the center panel in Fig. 2B. The mean and the covariance encode a distribution over trajectories in the feature space and through the relationship ***τ*** = **Φ*****w***, the distribution can be mapped (back) to the time domain. It is worth mentioning that a PTM can represent trajectories of varying lengths by substituting time by a movement phase. The last panel in Fig. 2B shows an illustrative example of a trajectory distribution and the corresponding input trajectories using a movement phase.

To conclude, the advantage of the presented time-series model is that it can be formulated as a generative probabilistic model for which many learning algorithms and similarity measures exist. The model can encode the signal variation and can be used to compute operations like predictions, model comparisons or trial likelihoods. These operations are explored in the present study on postural control with supportive contacts.

### Supportive contacts predict goal-directed movements

The learned and represented correlation of multiple limb trajectories can be exploited in computing predictions. This feature is used here to investigate if left wrist trajectories can predict the right wrist reaching trajectories. To separate the contributions of the trunk and the left arm, we transformed the wrist marker trajectories to a *shoulder frame*. For that the wrist positions in the subject’s coordinate frame were additionally corrected by the time-varying shoulder marker positions.

**Figure 3:**
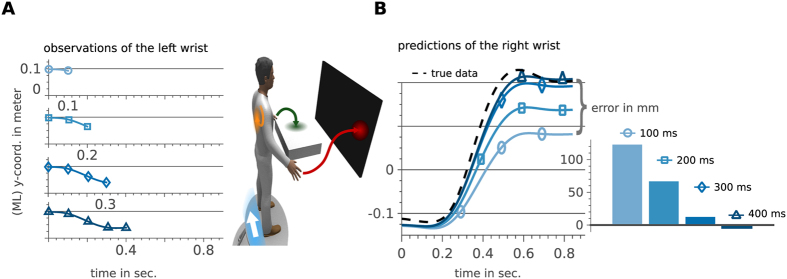
Supportive contacts predict goal-directed movements. (**A**) Partial observations of the left wrist predict right wrist future states in (**B**). For increasing observation horizons of the y-coordinate of the left wrist, the predicted right wrist trajectories converge to the true trajectory (illustrated as dashed line in (**B**). The final Euclidean error (to the true reached target on the screen) of less than 2 cm after 300 ms is 40 times smaller than the distance between the two outer targets (that is 80 cm).

**Figure 4:**
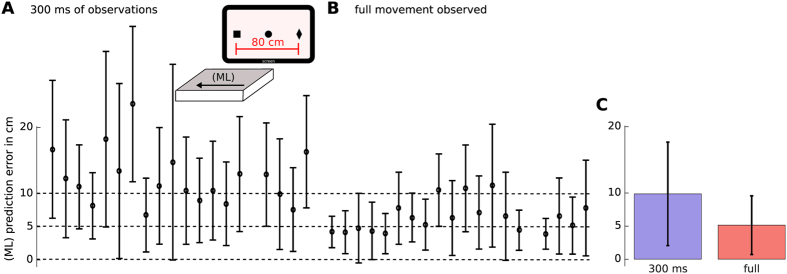
Target prediction error over all subjects. (**A**) Averaged prediction error for 19 subjects when observing 300 ms of the left wrist marker trajectories in perturbed trials. (**B**) The prediction error when observing the whole left wrist motion. Illustrated are the mean and the standard deviation over 18 test trials (six per target). One subject of 20 was excluded as less than six trials per target were recorded. Note that the maximum error or the distance between the two outer targets in the screen is 80 cm. (**C**) Mean and standard deviation over all subjects.

For increasing observation horizons up to 400 ms, exemplary predictions of the y-coordinate of the arm trajectories are shown in Fig. 3. For these examples the target prediction error was below 5 cm when observing only the first 300 ms of the left wrist motion. Note that the distance between the two exterior targets on the screen was 80 cm which defines the maximum error. Averaged over all subjects, the prediction error was 12.3 ± 9.8 cm for an observation horizon of 300 ms. When observing the complete trial the error was 6.4 ± 5.5 cm. Per subject errors are shown in Fig. 4. Note that to obtain these results we used 18 trials for training (trials 170–320) and 18 trials for testing (trials 321–400).

In summary, the PTM was used to train models of trajectory distributions that could predict the goal-directed reaching motions from observing solely the supportive left wrist movements. Accurate predictions at an early execution phase (i.e., the first 300 ms), where the effect of visual feedback is negligible, indicate that supportive contacts are at least partially pre-processed.

### Trunk adaptation precedes arm adaptation

**Figure 5:**
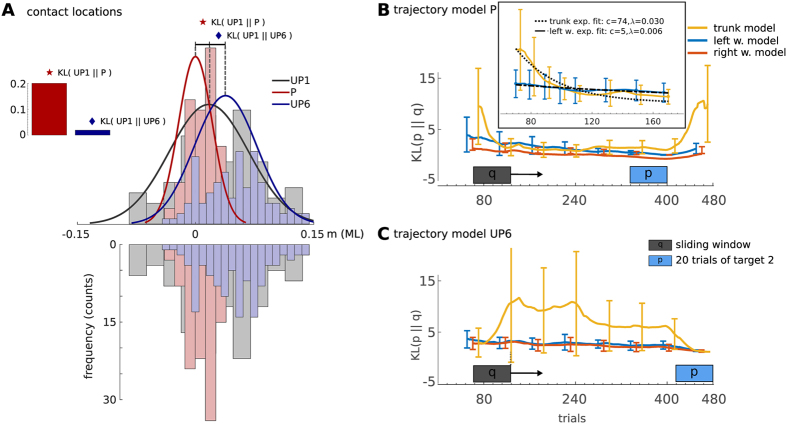
Trunk adaptation precedes arm adaptation. (**A**) The adaptation process of the contact location is illustrated by computing the KL-divergence between the first unperturbed session (UP1), the perturbed trials (P), and the last unperturbed session (UP6). The underlying data is shown as histogram with the Gaussian model fits as overlay. (**B**,**C**) For a detailed temporal analysis, the KL-divergence between a set of training trials (a sliding window of 20 trajectories) and a set of test trials (**B**) last 20 trials in P, (**C**) last 20 trials in UP6) is investigated. An exponential model fit is presented in the inset in (**B**).

To investigate adaptation of the supportive contacts we fitted Gaussian distributions to the contact locations recorded in the three phases of learning—the unperturbed training phase, the phase of perturbed trials and the final washout phase. Histograms over the contact locations in the shoulder frame are shown in Fig. 5A, where *UP1* denotes the initial training phase, *P* the perturbed trials and *UP6* the sixth session that was the washout phase. During the perturbed trials of the experiment, we found a shift of contacts to the right, or in other words, the left hand is moving closer to the center of the table in Fig. 1A. Note that the particular values in meters differ in Fig. 1A and in Fig. 5A. In the later the contacts are plotted in the shoulder frame.

The fitted Gaussian distributions in Fig. 5A indicate a symmetric shift of *UP6* with respect to *P*, which implies a possible after effect of perturbations. On the other hand, the reduced variance of UP6 with respect to UP1 and the overlap of both distributions suggest the increased precision of supportive hand contact at UP6 compared to UP1. To better understand this, we performed additional statistical analysis where we compared contact locations of UP1 and UP6. We found out that for two out of three targets, the contact location at UP1 was statistically different to the locations at UP6 (*t*(19) = −4.29 to −4.16, *p* < 0.001, *d* = −1.01 to −0.75) while for one target (right most) there was no difference between the contact locations (*t*(19) = 0.07, *p* = 0.948, *d* = 0.01).

To compare the similarities between the fitted Gaussian distributions for the three phases of learning, we used a Kullback-Leibler divergence (KL) distance measure. In line with the observation of the shift of contacts, we found strong similarities between the unperturbed phases, i.e., KL(UP1||UP6) = 0.019. In contrast for the transition to the perturbed trials we computed a distance of KL(UP1||P) = 0.20. For the transition to the washout phase, we found KL(P||UP6) = 0.126.

A more detailed investigation was conducted by analyzing trajectory similarities using the PTM. As reference model, trajectories of the last 20 trials in P or UP6 were used. We compared the reference model to a trajectory model trained from 20 trials in a moving window. The moving window traverses from trial 10 to trial 460 which is denoted by the shaded boxes in Fig. 5B,C. Individual models were trained for the left wrist, the right wrist and the trunk. We found that the trunk model converged about five times faster than the left wrist model. For that we used one-term exponential models of the form *y* = *c*exp(*λx*), where for the trunk *λ*_*t*_ = 0.03 and for the left wrist *λ*_*l*_ = 0.006. This result is highlighted in the inlay in Fig. 5B.

We found that the distribution over model parameters for generating trunk kinematics converges faster than the model for generating the arm kinematics. This is an potential indicator that postural control precedes volitional motor control. Our finding can be explained by the importance of correct trunk motions to prevent falling as observed in related work^31^,^36^. When comparing the prediction accuracy however, we made an interesting observation. During early phases of the motor execution the left wrist results in more accurate predictions. However, when observing the complete motion, the trunk was more informative for predicting the right arm movements, see Supplementary Figure S4. This result suggests that the supportive motion has a stronger effect on the task performance during early phases compared to the massive trunk.

## Discussion

It is known that supportive contacts aid postural control in humans^37^. Contacts increase the subjects’ belief about their poses in board balancing tasks^2^, in tasks without visual cues^32^, and have an effect on learning balancing strategies during task adaptation^6^. While these studies demonstrated the importance of contacts for human motor control, the contacts where *static* and pre-defined based on the experimental setting. In this study, we investigated the effect of the *active choice* of supportive contacts on motor control. Under natural conditions subjects were able to choose supportive contact locations to aid target reaching. Utilizing a developed probabilistic trajectory model (PTM), we found that the supportive motions leading to contacts could predict goal-directed reaching movements. A comparison of the rates of convergences of the model parameters in our PTM indicated that learning of postural control and arm reaching strategies proceeds on multiple time scales. These findings have important implications on medical care in diseases related to central nervous system disorders (such as dementia, Alzheimer’s, Parkinson’s disease or stroke), computational neuroscience and robotics.

### A pre-test for central nervous system disorders affecting postural control

Many central nervous system disorders affect not only cognitive abilities related to memory consolidation but also postural control. For example, an underdevelopment of postural control is a well known symptom in autism^38^,^39^. Early detection of these diseases is of utter importance for medical care. However, most pre-tests focus on cognitive functions that are influenced by many factors such as stress, sleep deprivation and age. Classical tests targeting motor coordination abilities are unnatural like for example the grooved pegboard test^40^, where the goal is to fit pegs of various shapes into punched holes. The presented experimental setting has the potential to become an alternative pre-test that investigates postural control and motor learning under natural conditions. Deficits in motor control can be quantified in terms of the model prediction performance as shown in Fig. 3. In the tested subjects, the average prediction error of the goal-directed target reaching motion was 6.4 ± 5.5(*SD*) cm out of a total range of 80 cm. Note that for these predictions the model input was solely the x,y,z coordinate of the supportive motion, see Fig. 4B. While the predictions were accurate for the tested healthy subjects, we speculate that participants with motor dysfunctions would show inferior prediction performance scores.

However, a limitation of the presented experimental setting is its simplicity. In our experiments the participants reached for a static target at one out of three possible locations on a horizontal line. Synchronization of movement onsets within 3–5 trials (see Fig. 1C,D) indicates that the task might be too simple to show significant effects in subjects with central nervous system disorders. More complex tasks could consider for example moving targets at arbitrary locations on the screen.

### Evidence for learning on multiple time scales

For many tasks motor memory consolidation proceeds on different time scales^25^,^41^,^42^. In particular, postural control is adapted on a faster rate in contrast to goal-directed movements. For example, Huys *et al*. showed that postural sway precedes eye and head movements (3:2 or also 3:1) in subjects learning to juggle^43^. In line with this finding, we found that trunk adaptation to maintain balance seems to precede the learning of optimal (here task correlated) supportive contact motions. Concretely, the learned generative model of trunk kinematics converged about 5 times faster than the model of the left wrist kinematics, see Fig. 5B. This result is another indicator for a hierarchical organization of motor control with the difference that we analyzed adaptation on a trajectory level in contrast to single time step models^19^,^25^,^41^. The developed PTM may extend future computational models for optimal feedback control by combining sequential predictions of feed forward commands and the integration of perceptual feedback.

A potential deficit of such an optimal feedback controller is that we analyzed adaptation on a kinematic level, where limb inertia has a delayed effect on the recorded marker trajectories. For example, while expressive motion vectors can be observed for the light weight arms, the massive trunk may have moved just for few millimeters which could result in inaccurate predictions. This hypothesis is confirmed by our results, where at early movement phases (up to 300 ms) the left wrist leads to more accurate predictions than the trunk, see Supplementary Figure S4. For observations of the complete trial however, the trunk motion is more informative. To avert the effect of limb inertia the PTM could be trained from Electromyography (EMG) patterns. Such models could be used to predict motor commands on a muscle level and could help to better understand the underlying motor control mechanisms.

### A robot controller that actively seeks for contacts

In this research we studied whole-body coordination mechanisms of arm reaching and postural control with additional hand contact. We investigated how humans perform reaching movements with the right arm in postural challenged conditions. Postural balance was maintained by establishing a supportive hand contact with the other arm. In effect, both arms had to perform reaching movements where one arm reaches for a distant target and the other arm reaches for a supportive hand contact. We found that humans preferred distinct contact locations for each of the three targets that were displayed. Such a context dependent controller could advance current abilities of humanoid robots as it was envisioned in the European project CoDyCo. In particular, our results suggest that different supportive contact strategies should be initiated based on future intentions. This has not been done so far as research on anticipatory controller focused largely on balancing^44^,^45^,^46^ or on compensating for external forces^47^,^48^,^49^,^50^.

In our experiments the modulating factors (the context) were the distance to the target, its location on the screen and the postural perturbation. The distance was chosen such that subjects *always* made a contact to avoid falling when reaching. We only investigated the effect of the contact location and used pre-defined translateral support base perturbations. The perturbations had no effect on the results. In unperturbed and perturbed conditions the subjects chose target dependent contact locations, see Fig. 1B. Future research may explore the effect of distance to complement a robot controller that autonomously decides in which situations to make supportive contacts.

## Methods

### Participants

Twenty right-handed male subjects (*age*: 20.8 ± 1.8(*SD*)) participated in the reaching experiments. None reported any neurological or musculoskeletal disorders (self-reported). Prior to their participation, the subjects were informed about the course of the study and were required to sign an informed consent approved by the National Medical Ethics Committee Slovenia (NO. 112/06/13). The experiments were carried out in accordance with the approved guidelines of the National Medical Ethics Committee Slovenia (NO. 112/06/13). All experimental protocol were approved by the National Medical Ethics Committee Slovenia (NO. 112/06/13).

### Apparatus

Participants stood on a force plate (9281CA, Kistler Instrumente AG, Winterthur, Switzerland) mounted on top of a Stewart platform^51^. The force plate was used to measure the six components of the ground reaction forces and torques to determine the center-of-pressure (CoP). The CoP was used to improve the quality of the automatically computed movement onsets of the left and the right wrist. In addition to a commonly used peak velocity criteria (two percent was used as threshold here) we ensured that the CoP velocity profiles exceeded a threshold of 0.001 meter per second. By that we could eliminate most trials with incorrectly computed movement onsets due to small movements of the wrists prior to the actual reaching motion. The correctness of the automatically computed movement onsets was verified through a visual inspection of the velocity profiles of all 9600 trials (480 trials × 20 subjects). Trials with incorrect movement onsets were either manually labeled or rejected.

A second force plate of the same type was mounted anteromedial to the subject’s hip position. In concrete, this force plate had an offset of Δ*x* = 0.42 m, Δ*y* = 0 m, and Δ*z* = 0.92 m with respect to the base. This force plate was used like a table providing additional support during reaching. The contact locations shown in Fig. 1 were defined as the left wrist position at the peak supportive contact force. In front to the contact force plate we mounted a screen. Both, the force plate and the screen were adjusted in height based on the subject’s trunk height (56.5 ± 2.4 cm).

The Stewart platform was used to apply translational perturbations in the mediolateral direction (ML). Specifically, the displacement of the platform was proportional to the sum of the anteroposterior (AP) components of the left and right hand displacements (denoted by Δ*x*_*LW*_ and Δ*x*_*RW*_). The maximal displacement of the Stewart platform was set to 0.2 m and corresponded to the situation when the subject’s left hand was at the far edge of the table and the finger on the right hand touched the screen, see Supplementary Figure S5. For this scenario the platform position in the (ML) direction was computed as *pos* = 0.1[Δ*x*_LW_/*d*_LW_ + Δ*x*_RW_/*d*_RW_], where *d*_LW_ = 0.68 m and *d*_RW_ = 1.12 m. For tracking the desired platform position a P-controller with a gain of *K*_*p*_ = 9 was used.

A motion capture system (NDI 3D Investigator) was used to track the participants wrists and trunk movements. Three motion tracking markers were attached to each wrist to compensate for occlusions during the reaching motions. At least one marker needed to be visible and for more than one visible marker the position was computed through averaging. Two markers were attached to the back at the scapulae to transform wrist trajectories to shoulder frames and to estimate the trunk motions (i.e., the center location of the two shoulder markers). Additional markers were placed at the screen, at a wearable thimble to estimate the right index fingertip position and at the force plate mounted on top of the Stewart platform.

### Coordinate frames

If not stated different, the results are defined in the subject’s coordinate frame. To compensate for the translational perturbations of the Stewart platform a marker was attached to the support force plate. The subjects coordinate frame was defined as the force plate marker position minus the initial left wrist location (to correct for small deviations of the subject’s feet placement throughout the experiment).

Wrist trajectories (see Fig. 3) were computed in the shoulder frames to isolate trunk and arm adaptations. In particular, the wrist positions in the subject’s coordinate frame were additionally corrected by the time-varying shoulder marker positions.

### Procedure

Participants had to perform 480 trials of reaching in blocks of 80 trials. After each block the subjects had a five minutes break. Only the first and the last 80 trials were unperturbed (see Fig. 1B). To investigate negative after effects, the perturbation was deactivated for 30 randomly selected reachings during trials 240 to 400. The participants were not informed about these catch trials.

Prior to each trial, subjects were required to stand upright moving as little as possible. A bar on the screen indicated anteroposterior fluctuations of the CoP in real time with respect to the initial CoP value. This initial CoP was measured at the beginning of the experiment. If the mean of the deviations averaged over 500 ms was less than 1 cm the start of the trial was initiated. After one second the target was presented at one out of three locations (target onset). The targets were displayed at a height of 1.5 m (z-coord. in Fig. 1A) and at a distance of 1.12 m (x-coord. in Fig. 1A). The y-coordinates of the three targets were −0.4, 0 and 0.4 meter. Note that three targets were chosen to define a non-trivial prediction problem that can be solved using the probabilistic trajectory model. In this case the trivial prediction in form of the mean over all kinematic trajectories would result in bad prediction performances for target one and target three.

After a random period (1–3 sec) the target’s color switched and the subjects were allowed to move. Movements prior to that visual cue terminated the trial. The target was reached if the distance between the fingertip and the target on the screen was less than 1 cm.

Participants received a monetary reward after each trial based on the time needed for reaching, i.e., EUR = 0.1–0.05*t* where *t* denotes the reaching time in seconds. The reaching time was defined as the difference between the target onset and the time until the target was reached. On average the subjects received five cents per trial which corresponds to a reaching duration of one second, see Supplementary Figure S6. If the target was missed, or if the target was not reached within two seconds, no reward was given.

### Data processing

All measurements of the force plates and the motion capture system were recorded at a rate of 100 Hz. Redundant marker settings on the wrists and on the thimble were used to compensate for occlusions. For that three markers were used for both wrists and the thimble. If all markers were occluded the trial was excluded from the analysis. For two or three correctly observed markers we computed the average for each coordinate (x,y,z). All marker trajectories were low-pass filtered at 5 Hz using 2nd-order Butterworth filter.

### Statistical tests

Statistical analyses were performed using SPSS 21 (SPSS Inc., Chicago, USA). For each subject, an average of left hand contact location in mediolateral direction was calculated for every combination of balance perturbation (unperturbed, perturbed and unperturbed catch trials) and target locations. The average values of the individual subjects were then used for statistical analysis. Effect of perturbation was investigated using one-way repeated measures ANOVA. Differences between perturbed and unperturbed sessions for each combination of independent variables were tested with post hoc t-tests with Bonferroni correction. The level of statistical significance was set to 0.05.

### Computing predictions through conditioning

Conditional probabilities and predictions are related concepts in statistical modeling. A prediction problem can modeled as computing the conditional probability denoted by


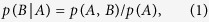


where *A* and *B* represent two random variables (RVs). In our experiment, the variable *A* could encode the probability of a certain contact location on the table and the target location is represented by the RV *B*. The joint distribution *p*(*A*, *B*) is the learned model and *p*(*A*) is a prior over all possible contact locations. Given a particular contact location *p*(*A* = *a*), we can compute the most likely target the human tries to reach. Throughout the paper, we will use the shorthand *p*(*a*) to denote *p*(*A* = *a*), which is the probability that the RV *A* takes the value *a* to keep the notation uncluttered.

A particular interesting class of distributions are Gaussian distributions


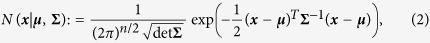


with the *n*-dimensional RV 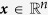, the mean ***μ*** and the variance **Σ**. In Gaussian distributions, conditional distributions can be computed in closed form. To see this, we define a multivariate normal distribution by concatenating two RVs. The two RVs denote the contact locations and the targets, i.e., 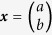, 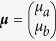 and 
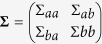
. Using these definitions in (2) and after rearranging the terms, the parameters *μ*_*b*|*a*_ and Σ_*b*|*a*_ of the conditional distribution can be computed as


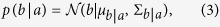



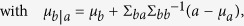



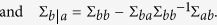


Given multiple measurements of *a* and *b* we can compute the statistics ***μ*** and **Σ**. For some during training unseen contact location a^*^ we can now predict the most likely target location (parametrized through 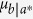 and 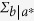). In the following we will extend this powerful feature of computing predictions to time-series data or trajectories.

### Phase modulated probabilistic models of trajectories

The same conditioning operation can be used for computing predictions in trajectories if the RVs encode feature vectors in function approximation, see Fig. 2 for a sketch. Let 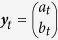 denote a concatenated state at time *t* representing the contact location and the target. Only for the sake of clarity we will again assume that the contact location and the target are scalars. As we will see later the results generalize to multi-dimensional states. In addition, we will assume for simplicity that each dimension in ***y***_*t*_ is approximated through a *J*-dimensional feature vector 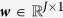 and basis function vectors 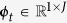, e.g. *a*_*t*_ = ***ϕ***_*t*_***w***_*a*_ with ***ϕ***_*t*_ = [*ϕ*_*t*,1_,..., *ϕ*_*t*,*J*_]^*T*^. A large variety of possible basis functions can be used for time series approximation. A popular choice for rhythmic movements are Von-Mises basis functions, whereas Gaussian basis functions are widely used for point to point movements^52^,^53^. In this study we used Gaussian basis functions with


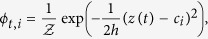


where 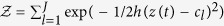 denotes a normalization term. The function *z*(*t*) implements a mapping from discrete time steps to a movement phase, i.e., *z*(*t*) = (*t* − 1)/(*T* − 1) with 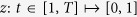. In this notation state sequences of different length can be modeled and are aligned through the movement phase. Note that in our reaching experiments *z*(*t*) = 0 denotes the movement onset and *z*(*t*) = 1 the event when the target was reached. The scalar *c*_*i*_ ∈ [0, 1] denotes the center of the *i*-th basis function and *h* is the bandwidth parameter.

In general, *D*-dimensional states can be approximated in ***y***_*t*_ = **Φ**_*t*_ ***w*** using block diagonal matrices 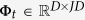 and concatenated feature vectors 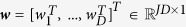. For example, when modeling contacts and targets, 
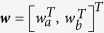
 and a block diagonal matrix of the form 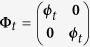 is used.

Sequences of *T* states, denoted by ***τ*** = ***y***_1:*T*_, can be compactly represented by


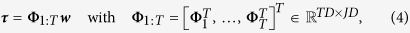


where **Φ**_1:*T*_ denotes an extended basis function matrix. Using the function approximation in (4) we can define the generative probabilistic model for trajectories


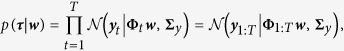


where **Σ**_*y*_ models zero mean independent and identically distributed (i.i.d.) Gaussian noise in ***y***_*t*_ = **Φ**_*t*_ ***w*** + ***ε***_*y*_ with 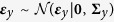. In order to represent a distribution over trajectories *p*(***τ***), we can apply the product rule shown in (1) and compute the marginal. For Gaussian distributions, in particular for 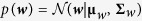, the marginal can be computed in closed form


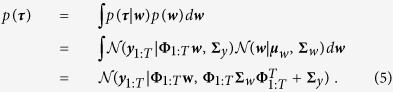


The mean ***μ***_*w*_ and the covariance matrix **Σ**_*w*_ can be learned from data by maximum likelihood using the Expectation Maximization (EM) algorithm^54^. A simpler solution that works well in practice is to compute first the most likely estim ate of ***w***^[*i*]^ for each trajectory ***τ***^[*i*]^ independently, where the index *i* denotes the *i*-th demonstration. In particular, given the trajectory ***τ***^[*i*]^, the corresponding weight vector ***w***^[*i*]^ can be estimated by a straight forward least squares estimate


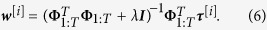


Note that the least squares estimate is also known as regularized pseudo-inverse solution to the generative model ***τ***^[*i*]^ = **Φ**_1:*T*_***w***^[*i*]^. The regularization parameter *λ* is used to avoid numerical singularities. In our recordings we used *λ* = 1*e* − 6. After computing the vectors for each trajectory, the mean and the covariance of *p*(***w***) can be estimated by the sample mean and sample covariance of the ***w***^[*i*]^ vectors.

### Computing predictions in trajectories

Let us assume that we have observed a sequence of states 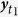 to 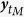 at *m* = 1, 2,..., *M* different time points, which do not need to be sampled at uniform intervals. In addition, not all of the dimensions in the vector 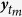 might be observed (e.g. due to occlusions, where irrelevant dimensions are assumed to be set to some real number). We introduce the homogeneous covariance matrix **Σ**_***o***_ to control the importance of the observations, where small diagonal variances denote important dimensions and large scalars irrelevant dimensions. For example, we would use 
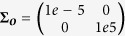
 if only the first out of two dimensions is observed (as in the example with contact locations and targets). Let us further denote **Φ**_***o***_ as the concatenation of the basis function matrices for these time points and ***o*** as concatenation of the 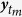 vectors. Given these observations, we can obtain a conditioned distribution *p*(***w***|***o***) over the weight vectors


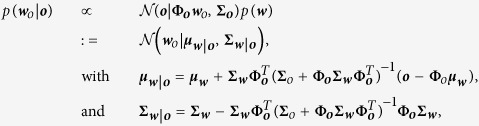


which recovers the conditioning result in (3) in the feature space.

To predict the state sequence 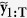, the conditional distribution is projected back into the trajectory space using (5). The result is a distribution over trajectories


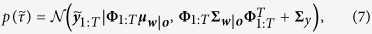


where for *t*_*M*_ < *T* in the observations 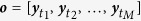 future states can be predicted. For details on the implementation and usage of the model we refer the reader to the Supplementary Code to this manuscript^55^.

### Computing model comparisons in trajectories

Using the proposed probabilistic trajectory model (PTM), two trajectory models can be compared through computing the Kullback-Leibler (KL) divergence. For Gaussian distributions as in (5) the KL divergence can be computed in closed form


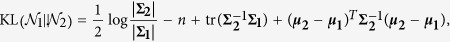


where 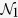 denotes a Gaussian with the *n*-dimensional mean ***μ***_**1**_ and the covariance **Σ**_**1**_. The symbol tr denotes the matrix trace.

### Computing task likelihoods in trajectories

Another important probabilistic operation is the computation of the task likelihood. This operation can be used to test which candidate model out of several best explains the data. Let 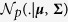 denote the model under test. Given a single trajectory sample τ^*^ and its feature space representation w^*^ we can compute


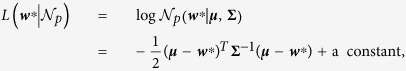


which quantifies how likely τ^*^ = **Φ**_1:*T*_w^*^ was generated by model 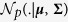.

## Additional Information

**How to cite this article**: Rueckert, E. *et al*. Probabilistic Movement Models Show that Postural Control Precedes and Predicts Volitional Motor Control. *Sci. Rep.* **6**, 28455; doi: 10.1038/srep28455 (2016).

## Supplementary Material

Supplementary Information
