# Author Correction: Probabilistic Movement Models Show that Postural Control Precedes and Predicts Volitional Motor Control

**DOI:** 10.1038/s41598-020-63129-x

**Published:** 2020-04-16

**Authors:** Elmar Rueckert, Jernej Čamernik, Jan Peters, Jan Babič

**Affiliations:** 1 Intelligent Autonomous Systems Lab, Technische Universität Darmstadt, Darmstadt, 64289 Germany; 2 Department for Automation, Biocybernetics and Robotics, Jožef Stefan Institute, Ljubljana, SI-1000 Slovenia; 3 Robot Learning Group, Max-Planck Institute for Intelligent Systems, Tuebingen, 72076 Germany; 4 Jožef Stefan International Postgraduate School, Jamova cesta 39, 1000 Ljubljana, Slovenia

Correction to: *Scientific Reports* 10.1038/srep28455, published online 22 June 2016

This Article contains an error in the affiliation list, where an affiliation for Jernej Čamernik is missing. The correct affiliations for Jernej Čamernik are listed below:

Department for Automation, Biocybernetics and Robotics, Jožef Stefan Institute, Ljubljana, SI-1000, Slovenia

Jožef Stefan International Postgraduate School, Jamova cesta 39, 1000 Ljubljana, Slovenia

