# Supplementary material for: Probabilistic Movement Models Show that Postural Control Precedes and Predicts Volitional Motor Control
Source: Sci Rep. 2016 Jun 22;6:28455. doi: 10.1038/srep28455 (PMC4916463; doi:10.1038/srep28455)
Supplement: Supplementary Information [file srep28455-s1.pdf]

Supplement to *Probabilistic Movement Models Show that Postural Control Precedes and Predicts Volitional Motor Control*

Elmar Rueckert[1,*], Jernej Čamernik[2], Jan Peters[1,3], and Jan Babič[2]

[1]Intelligent Autonomous Systems Lab, Technische Universität Darmstadt, 64289, Germany
[2]Department for Automation, Biocybernetics and Robotics, Jožef Stefan Institute, Ljubljana, SI-1000, Slovenia
[3]Robot Learning Group, Max-Planck Institute for Intelligent Systems, Tuebingen, 72076, Germany
[*]rueckert@ias.tu-darmstadt.de

ABSTRACT

This supplement provides additional figures to the human recordings (Fig. S1, S2), the probabilistic trajectory model (Fig. S3, S4) and the experimental setting (Fig. S5, S6).

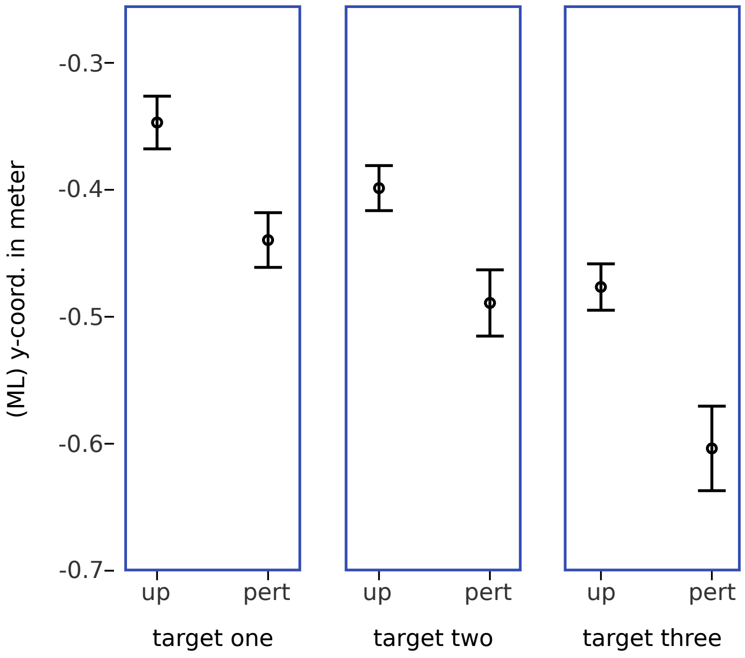

Figure S1. Supplementary figure, Means of the contact locations: From left to right the means and the standard deviations over all 20 subjects are shown for the three target locations. In each panel we contrast the unperturbed and the perturbed session.

Details to Supplementary Figure S7 The primary role of the monetary reward was to motivate the subjects to perform fast reaching movements. By that we could study skill adaptation within the first few hundred milliseconds where feedback had no effect. Here we provide additional results that show that the target location had an effect on the reward but not the experimental condition (unperturbed or perturbed).

Analysis of variance (ANOVA) showed significant effect in the amount of monetary reward between sessions when the subjects were perturbed and unperturbed during reaching toward the targets, $F(1,19) = 8.581$, $p = .009$, $\eta_p^2 = .311$.

Post hoc analyses showed that the amount of received reward was significantly different between reaching toward all three distinctly positioned targets in unperturbed trials,$t(19) = -15.44$ to 11.77, $p \leq .038$, and between targets 1 and 2 and targets 2 and 3, $t(19) = -15.51$ to -12.51, $p < .001$ in perturbed trials.

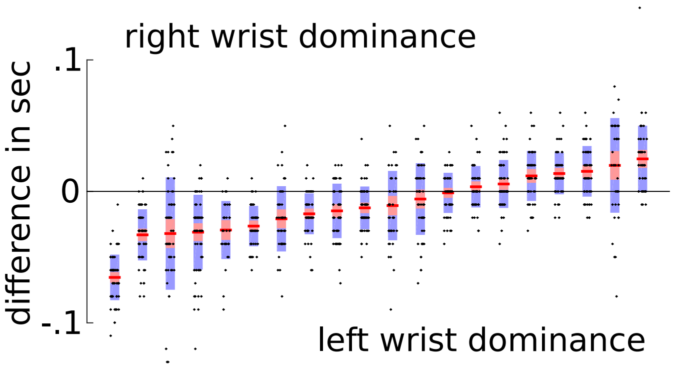

Figure S2. **Supplementary figure, Lead of the left wrist in 12 out of 20 subjects:** We show the difference of the movement onsets (left wrist minus right wrist). The underlying data are representative trials of the 2nd perturbed session (170 to 240), where learning converged.

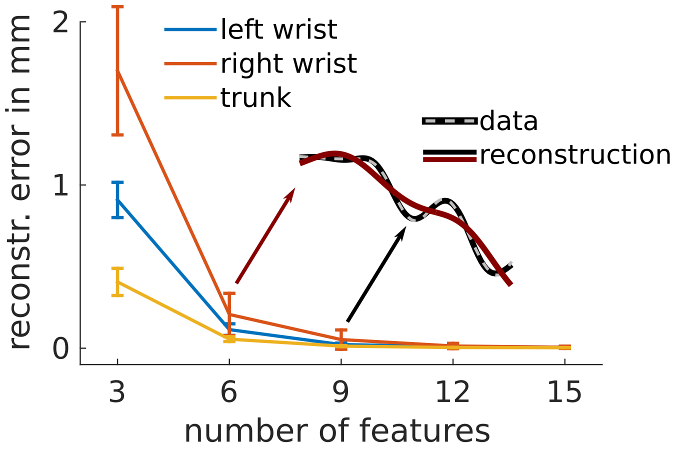

Figure S3. **Supplementary figure. Model complexity (number of basis functions) determines the reconstruction error:** With an increasing number of basis functions the reconstruction error (shown in millimeter) decreases. The reconstruction error is defined as the Euclidean distance of the generated trajectory to its observed counterpart. For the three limbs, left wrist, right wrist and trunk, the error converges to zero with more than nine basis functions. Ten basis functions are used in the experiments in the manuscript.

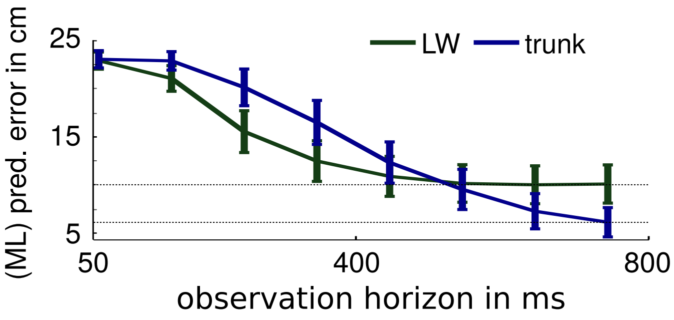

Figure S4. **Supplementary figure, Trunk vs. Left Wrist prediction error:** Average prediction error in cm over all subjects given the left wrist (LW) or the trunk motion as observation.

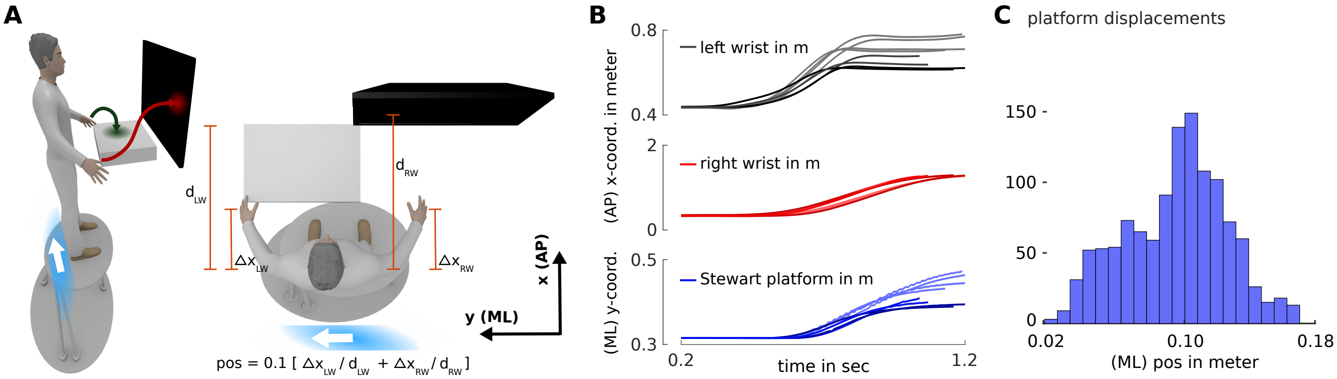

Figure S5. **Supplementary figure, Translateral perturbations: A)** The Stewart platform was used to apply translational perturbations in the mediolateral direction. Specifically, the displacement of the platform was proportional to the sum of the anteroposterior components of the left and right hand displacements. The maximal displacement of the Stewart platform was 20 cm and corresponded to the situation when the subject's left hand was at the far edge of the table and the finger on the right hand touched the screen. **B)** Sample trajectories of the left wrist, the right wrist and the displacement of the platform.

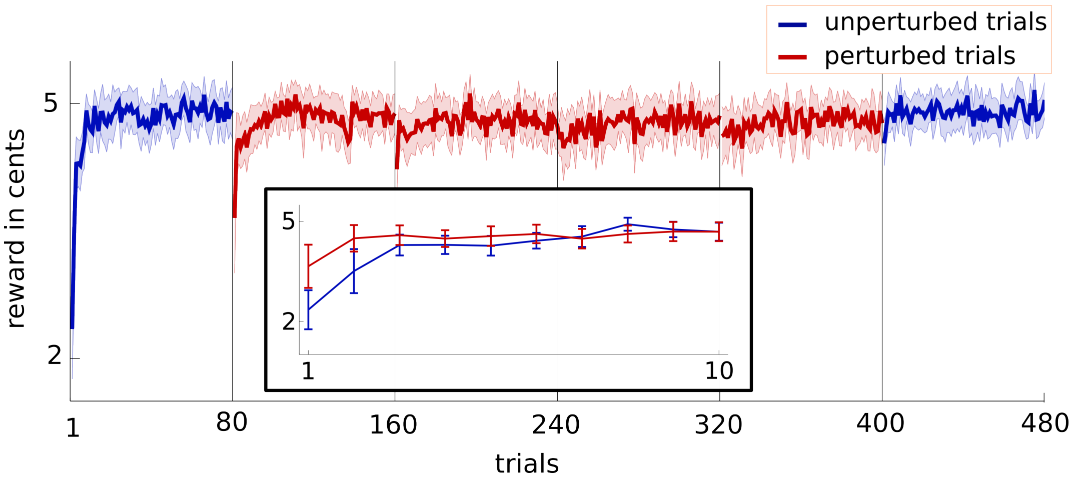

Figure S6. **Supplementary figure, Monetary rewards received:** Illustration of the received rewards averaged over all 20 subjects. The shaded region denotes the confidence bound.

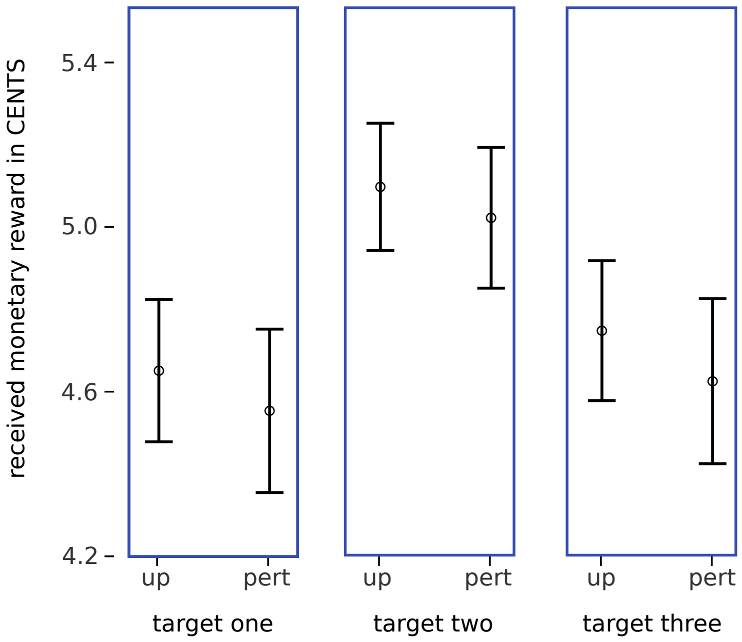

Figure S7. Supplementary figure, Means of monetary rewards received: From left to right the means and the standard deviations over all 20 subjects are shown for the three target locations. In each panel we contrast the unperturbed and the perturbed session.

However, the post hoc analysis showed that there were no significant differences between means of received reward for each target between perturbed and unperturbed trials (target one, $t(19) = 3.42$, $p = .057$; target two, $t(19) = 2.9$, $p = .171$; target three, $t(19) = 3.47$, $p = .057$).
